# Lifestyle Composite and Resilience to Alzheimer's Disease Pathology in Down Syndrome

**DOI:** 10.1111/jar.70109

**Published:** 2025-08-13

**Authors:** Emily K. Schworer, Matthew D. Zammit, Benjamin L. Handen, Brianna Piro‐Gambetti, Melissa R. Jenkins, Courtney Brothers, Ozioma C. Okonkwo, Christy L. Hom, Beau M. Ances, Bradley T. Christian, Sigan L. Hartley

**Affiliations:** ^1^ Waisman Center University of Wisconsin‐Madison Madison Wisconsin USA; ^2^ Department of Psychiatry University of Pittsburgh Pittsburgh Pennsylvania USA; ^3^ Alzheimer's Disease Research Center University of Wisconsin‐Madison Madison Wisconsin USA; ^4^ Department of Psychiatry and Human Behavior University of California, Irvine Orange California USA; ^5^ Department of Neurology Washington University in St. Louis St. Louis Missouri USA

**Keywords:** amyloid‐beta, cognition, employment, leisure, physical activity

## Abstract

**Background:**

People with Down syndrome (DS) have a high risk for Alzheimer's disease (AD). Identifying resiliency factors for AD is of critical importance to the DS community.

**Method:**

Participants were 63 adults with DS. Measures included amyloid‐beta PET scans (amyloid age), National Task Group‐Early Detection Screen for Dementia (NTG‐EDSD), and Down Syndrome Mental Status Examination (DSMSE). Lifestyle composites were created by assessing time spent in leisure, employment, and physical activity across 7 days through informant reports and accelerometry.

**Results:**

There was a significant moderation effect of the lifestyle composite on the association between amyloid age and the NTG‐EDSD and DSMSE. Participants with a higher lifestyle composite (higher leisure, employment engagement, and physical activity) had fewer dementia symptoms than those with a lower lifestyle composite score of a similar amyloid age.

**Conclusions:**

Modifiable lifestyle factors may allow adults with DS to maintain cognitive functioning for longer in the face of AD pathology.

**Summary**

- Lifestyle factors can improve cognitive resilience in people with Down syndrome; more (versus less) engagement in leisure activities, employment‐type activities (paid or non‐paid), and physical activity were associated with less cognitive impairment given a similar level of Alzheimer's disease pathology in the brain.
- Modifiable lifestyle factors may allow adults with Down syndrome to maintain cognitive functioning for longer in the face of early Alzheimer's disease pathology.
- Findings are an important first step in identifying lifestyle intervention targets to promote healthy ageing in individuals with Down syndrome.

Trisomy 21 causes Down syndrome (DS) and is a genetic risk factor for Alzheimer's disease (AD) (Fortea et al. 2021; Wiseman et al. 2015), a progressive neurodegenerative disease involving declines in memory and thinking skills and eventually the loss of everyday living skills (Alzheimer's Association, 2024; American Psychiatric Association 2013). It is estimated that 50% of adults with DS develop symptomatic AD by age 53% and 90% within their lifetime (Iulita et al. 2022; McCarron et al. 2017). The high risk for DSAD is due to an overproduction of amyloid‐beta (hereon referred to as amyloid), driven by the triplication of the amyloid precursor protein (*APP*) located on chromosome 21 (Antonarakis et al. 2020). Early AD pathology, in the form of amyloid plaques, can be detected through neuroimaging decades prior to clinical symptoms of DSAD (Lott and Head 2019; Zigman and Lott 2007), similar to autosomal dominant AD (Bateman et al. 2012) and sporadic late‐onset AD (Schindler et al. 2021). Outside of DS, lifestyle factors including leisure activities, employment engagement, and physical activity can reduce the risk of AD and/or delay the onset of symptoms in the face of AD pathology (Galvin et al. 2021; Livingston et al. 2024; Lupton et al. 2010; Okonkwo et al. 2014). Lifestyle factors are often interrelated (i.e., the same individual engages in multiple beneficial lifestyle behaviors) and can have synergistic protective effects on cognitive aging and AD (Dhana et al. 2020; Dhana et al. 2022; Sabia et al. 2009). Indeed, composite scores that draw on multiple unhealthy lifestyle factors are stronger predictors of aging‐related cognitive impairments (odds ratio 2.52–2.87) than lifestyle factors in isolation (odds ratios 0.94–2.38) (Sabia et al. 2009). In DS, lifestyle factors are individually associated with cognitive functioning (Fleming et al. 2021; Piro‐Gambetti et al. 2024) and amyloid burden in adulthood (Mihaila et al. 2019), but their combined protective effect on DSAD has not been studied. The current study examined the effect of a lifestyle composite (based on leisure, employment engagement, and physical activity) on the association between amyloid age (i.e., estimated years to or from evidencing elevated Aβ) and AD‐related cognitive impairments in adults with DS.

Efforts to identify factors that promote resilience in DSAD and could be targets of intervention or policy aimed at encouraging healthy aging have been identified as critical to the DS community (National Institutes of Health 2022). In autosomal dominant AD and sporadic late‐onset AD, resilience is defined as the delay of cognitive or functional decline in the face of early AD pathology (Arenaza‐Urquijo and Vemuri 2018). Resilience mechanisms are theorized to operate by contributing to the development of neurobiological capital prior to AD pathology, helping maintain brain structure and function in the presence of AD pathology, and/or fostering cognitive strategies to compensate for declines in cognitive abilities (cognitive reserve) (Arenaza‐Urquijo and Vemuri 2018; Arenaza‐Urquijo and Vemuri 2020; Stern 2012). Outside of DS, several lifestyle factors have been shown to build resilience to AD, such as leisure, employment, and physical activity (Clare et al. 2017; Song et al. 2022); however, little research has examined the combined benefit of these lifestyle factors for building resilience to DSAD.

A growing number of studies on the adult general population have reported benefits of leisure activities, particularly those that involve cognitive stimulation (e.g., puzzles, games, learning) or social engagement (e.g., outings with friends or social clubs), for reducing the risk of AD or delaying cognitive decline in the presence of early AD pathology (Galvin et al. 2021; Wang et al. 2013). While the causal mechanisms are not definitive, cognitive and social leisure activities are thought to promote lifelong synapse development (Heckman 2008), build cognitive reserve by developing executive functioning skills, and aid in brain maintenance by reducing stress and inflammation and preventing white matter loss (Duffner et al. 2022; Sommerlad et al. 2023). Recently, there has been attention to the potential benefits of cognitive and social leisure for adults with DS as they age (Carr 2008; Lifshitz‐Vahav et al. 2016; Mihaila et al. 2019; Mihaila et al. 2017). For example, engaging in more cognitively stimulating and recreational activities was associated with better cognitive performance in 25 to 55‐yearyear‐old adults with DS when controlling for chronological and mental age (Lifshitz‐Vahav et al. 2016). Moreover, adults with DS aged 30 to 53 who engaged in more (versus less) social leisure activity exhibited less decline in episodic memory three years later (Mihaila et al. 2019). This same study also found that leisure activity moderated the relation between amyloid accumulation and decline in cognitive function over this 3‐year period (Mihaila et al. 2019). Together, these findings suggest that cognitive and social leisure engagement may attenuate the effects of early amyloid burden on cognitive decline in DSAD.

Occupation and employment activities have also been associated with delayed onset of AD dementia in the general population (Lupton et al. 2010). Later age of retirement is associated with a later age of AD diagnosis (Grotz et al. 2015). Employment is thought to impact brain maintenance and cognitive reserve largely through cognitively stimulating activities such as requiring individuals to learn new skills, problem‐solve, and think flexibly at a daily level (Vance et al. 2016). Within DS, less is known about the association between employment and DSAD. One study found associations between the type and complexity of work and memory decline (Piro‐Gambetti et al. 2024). Specifically, adults with DS who engaged in more complex work that involved the manipulation of objects and social interactions evidenced less cognitive decline over 3 years in models controlling for intellectual disability level. Given that over half of adults with DS in the United States have paid or volunteer jobs (Kumin and Schoenbrodt 2016), investigating the potential resilience effect of cognitively stimulating employment activities that promote learning and problem‐solving in DSAD is important.

In both typically developing adults and those with DS, there is evidence that physical activity reduces cognitive declines associated with aging and promotes brain health (Fleming et al. 2021; Okonkwo et al. 2014). For example, older adults in the general population who engage in more physical activity have lower amyloid levels (Brown et al. 2013). Physical activity has also been a target for healthy aging interventions. A randomized control trial in typically developing older adults found some improvements in cognition after a 6‐month home‐based physical activity program aimed to increase physical activity to 150 min per week (Lautenschlager et al. 2008). Within DS, spending more time in sedentary behaviour is associated with dementia symptoms (Fleming et al. 2021), while engaging in more physical activity is related to better memory, executive function, visuospatial, and adaptive performance (Fleming et al. 2021; Pape et al. 2021; Peven et al. 2022). Additionally, short‐term cognitive improvements were seen after a 12‐week exercise intervention involving adults with DS (Ptomey et al. 2018). These intervention findings, along with the empirical findings of connections between more physical activity and better cognitive functioning across adulthood, highlight the potential resilience effect of physical activity for DSAD.

The current study investigated the combined effect of three lifestyle factors—leisure, employment engagement, and physical activity—on the association between amyloid age and AD‐related cognitive decline in adults with DS. Amyloid age was used in place of centiloid values, as the amyloid age variable places individuals' amyloid accumulation on a scale in years to improve interpretability and provides a time estimate to clinical symptom onset (Zammit et al. 2024; Schworer, Zammit, et al. 2024). In line with theory (Arenaza‐Urquijo and Vemuri 2020) and evidence outside of DS (Dhana et al. 2020; Galvin et al. 2021), the lifestyle composite was expected to moderate the association between amyloid age and cognitive performance. Specifically, we hypothesised that adults with DS with a higher lifestyle composite (i.e., engaged in more cognitive and social leisure, employment that promotes problem‐solving and learning, and physical activity) would exhibit fewer dementia symptoms given a similar amyloid age than adults with DS with a lower lifestyle composite score. In addition, the moderation effect of the lifestyle composite score was hypothesised to be stronger than the moderation effect of each lifestyle factor alone (leisure, employment, and physical activity).

## 1 Method

### 1.1 Participants

Participants were 63 adults with DS aged 28–59 years old. Adults in the study were recruited from the Alzheimer Biomarker Consortium‐Down Syndrome (ABC‐DS; Handen et al. 2020). To be included in the study, participants were required to be ≥ 25 years old, have genetic testing results indicating trisomy 21 (full, mosaic, or translocation), have a mental age above 30 months, and not have any untreated health conditions that could impact neuropsychological testing. The majority of participants were cognitively stable (84.2%); 9.5% had mild cognitive impairment, and 6.3% had dementia. Dementia status was determined through consensus meetings involving a psychologist, physician, and at least two other team members (Handen et al. 2020). ID level was determined using abbreviated battery IQ scores from the Stanford‐Binet, fifth edition or KBIT‐2 administered prior to having any concerns about dementia. The lowest possible standardized IQ score on these is 47 or 40, which does not allow us to differentiate moderate from severe/profound ID. Thus, we used mental age equivalent scores to categorise ID level as: mild: 9–14 years, moderate: 4–8 years, and severe/profound: ≤ 3 years. See Table 1 for participant demographics.

**TABLE 1:** Descriptive statistics for participant demographics and lifestyle factors, *n* = 63.

	M (SD)/*n* (%)
Chronological age	40.21 (7.73)
*Sex*, *n* (%) male	35 (55.6%)
Race	
White	62 (98.4%)
Mixed race	1 (1.6%)
Ethnicity	
Non‐Hispanic	62 (98.4%)
Hispanic	1 (1.6%)
Down syndrome type	
Trisomy 21	60 (95.2%)
Translocation	3 (4.8%)
Lifetime ID level	
Mild	32 (50.8%)
Moderate	22 (34.9%)
Severe	9 (14.3%)
Dementia symptoms	
NTG‐EDSD total score	5.00 (8.85)
DSMSE total score	63.68 (16.45)
Lifestyle factors	
Cognitive leisure frequency	44.21 (16.06)
Social leisure frequency	12.51 (6.71)
Employment activity frequency	8.35 (5.30)
Physical activity (avg. steps per day)	11091.97 (4210.81)

*Note:* ID = Intellectual disability; NTG‐EDSD = National Task Group–Early Detection and Screening for Dementia; DSMSE = Down Syndrome Mental Status Examination.

* *p* < 0.05.

** *p* < 0.01.

### 1.2 Procedure

Study procedures were completed across multi‐day study visits to one of four ABC‐DS sites that were part of an auxiliary study on lifestyle risk and resiliency factors. Participants with DS were accompanied to the study visit by a study partner (parent, sibling, or caregiver) who knew them well. Study partners completed questionnaires and interviews about the participant with DS's medical history, behavioural and cognitive functioning, and lifestyle. The participant with DS completed a neuropsychological battery and underwent a blood draw and imaging procedures involving magnetic resonance imaging (MRI) and positron emission tomography (PET) scans. At the conclusion of the in‐person study visit, the participant with DS was sent home with an actigraphy watch and daily diary to record information about daily activities including physical activity for one week.

### 1.3 Measures

#### 1.3.1 Amyloid Age

Structural T1‐weighted MRI scans were completed and processed using FreeSurfer v5.3.0. Participants also completed an Aβ PET scan using [^11^C] Pittsburgh Compound‐B (PiB). For more detail on imaging collection and processing methods see Zammit et al. (2024). Aβ trajectories (in Centiloids) were modeled using a sampled iterative local approximation (SILA) algorithm to assign each individual an amyloid age value, representing the duration of Aβ positivity in years (Zammit et al. 2024; Schworer, Zammit, et al. 2024). Amyloid age values were centered at 18 Centiloids, the point at which an individual is considered to have Aβ positivity. Aβ negative individuals display negative amyloid age values, representing the estimated number of years to Aβ positivity, while Aβ positive individuals displayed positive amyloid age values, indicative of the number of years since reaching Aβ positivity.

#### 1.3.2 Dementia Symptoms and Cognitive Abilities

Study partners completed the National Task Group‐Early Detection Screen for Dementia (NTG‐EDSD) (Esralew et al. 2018), a questionnaire used to assess functional and behavioral dementia‐related changes in individuals with intellectual and developmental disabilities. Responses from study partners of “always been the case” and “does not apply” are coded as 0 and responses of “always but worse” and “new symptom in past year” are coded as one and summed within each domain. The 6‐domain total score of the scale, comprising of activities of daily living, language and communication, sleep–wake change patterns, ambulation, memory, and behavior and affect subdomains, ranges from 0 to 51, with higher scores indicating more dementia symptoms. The NTG‐EDSD is a sensitive screening tool for MCI and AD in adults with DS (Silverman et al. 2021).

Participants also completed the Down Syndrome Mental Status Examination (DSMSE) (Haxby 1989). The DSMSE includes domains of knowledge, memory, apraxia, language, and visuospatial reasoning and has demonstrated diagnostic utility for distinguishing adults with DS and AD dementia from cognitively stable adults with DS (Krinsky‐McHale et al. 2020). Higher scores indicate better cognitive performance (range 0 to 87).

#### 1.3.3 Lifestyle Factors

**FIGURE 1:**
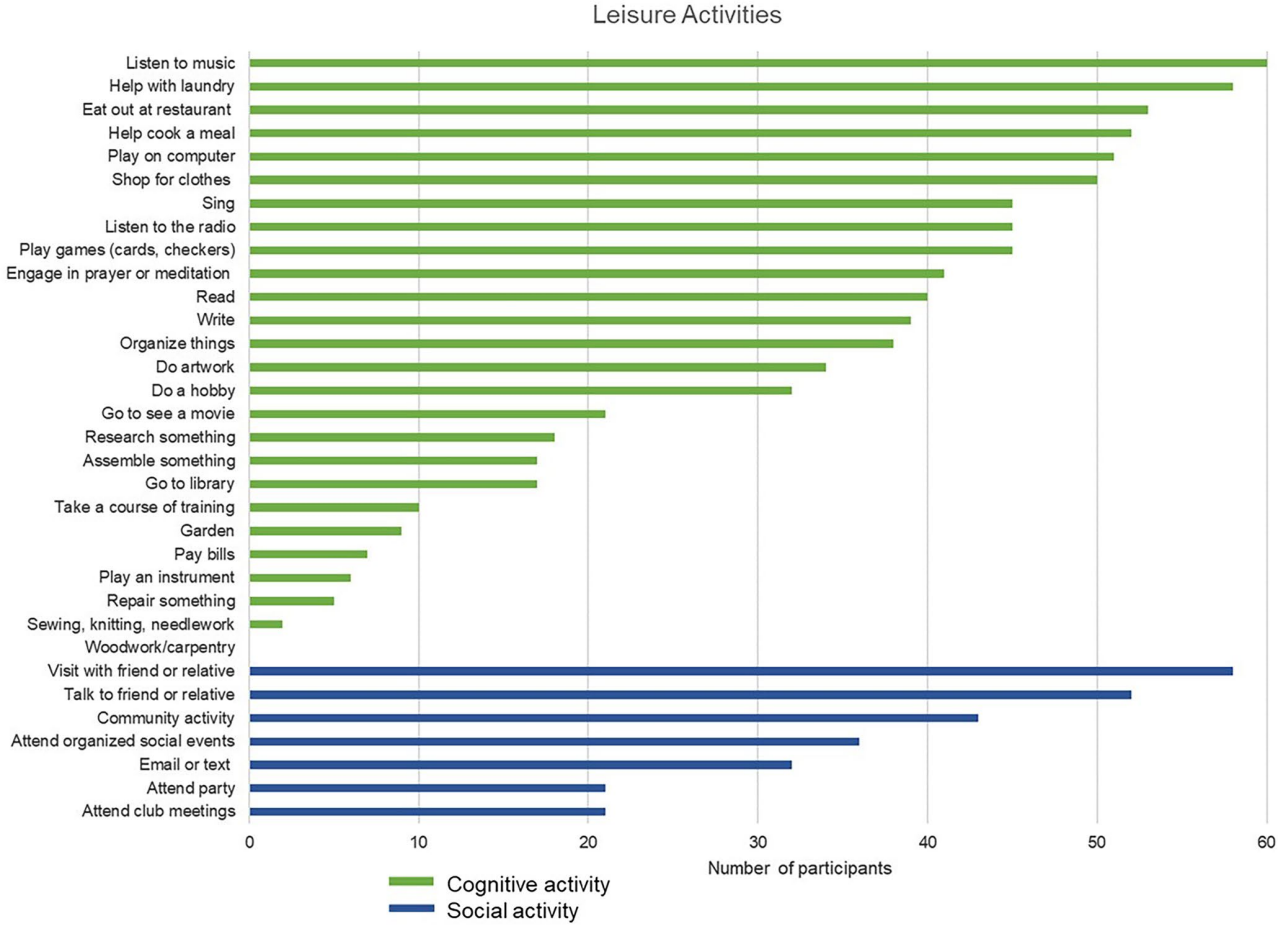
Cognitive and social leisure activities completed by adults with DS at least once a month.

Cognitive and social leisure activity (modified from Jopp and Hertzog 2010). Study partners rated the amount of time the adult with DS participated in 33 types of leisure activities on a scale from 0 (less than once a month) to 5 (daily) (activities listed in Figure 1). The study partner rating for each endorsed activity was summed across the items to create a total score (higher = more involvement in leisure). Leisure activities were separated into those that provided cognitive stimulation (26 activities) and those that fostered social engagement (7 activities). Similar lists of leisure activities and separation of social and cognitive leisure have been used in previous studies that included adults with DS (Mihaila et al. 2019). Number of activities endorsed and a frequency score (sum of Likert scale ratings) are reported for both cognitive and social leisure.

### 1.4 Employment Activity

**FIGURE 2:**
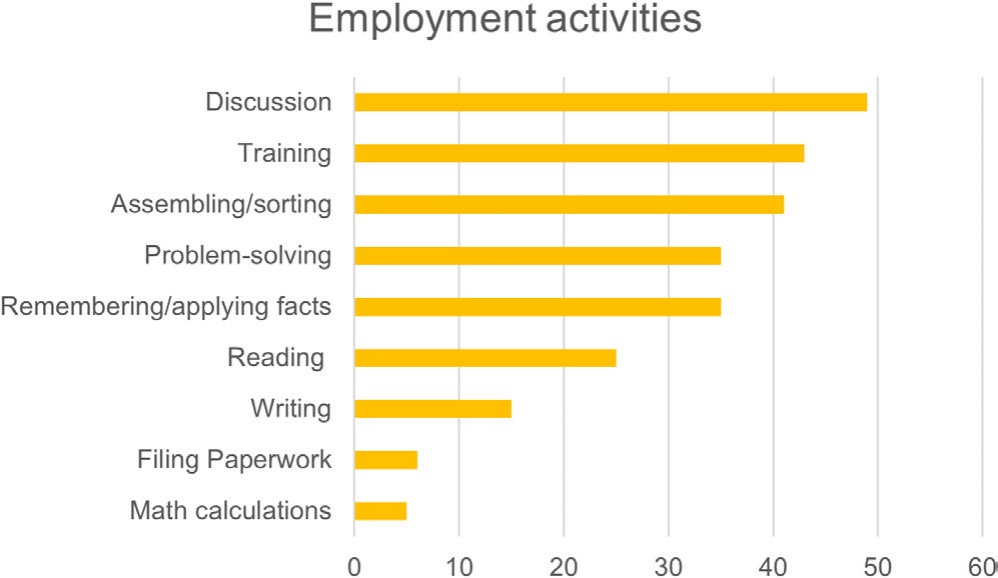
Number of adults with DS participating in each cognitively stimulating employment activity.

Study partners were asked to rate the amount of time the adult with DS participated in nine types of cognitively stimulating vocational activities on a scale from 0 (little to none) to 5 (more than 2 h) on a typical work day (activities listed in Figure 2). The study partner rating for each endorsed activity was summed across the items. Higher scores indicated more involvement in cognitively stimulating employment activities. The number of employment activities endorsed and a frequency score (sum of Likert scale ratings) are reported.

### 1.5 Physical Activity

Participants wore a GT9X Actigraph accelerometer for seven days on their nondominant wrist to assess physical activity during awake hours. To be included in the analysis, participants were required to wear the actigraphy watch for at least three days, and there needed to be 400 or more minutes of wear time each day. Step count was calculated by the device, and average daily step count was used to estimate participants' physical activity (Peven et al. 2022; Trost and O'Neil 2014).

### 1.6 Analysis Plan

Descriptive statistics were used to describe cognitive and social leisure, employment, and physical activity in the sample of adults with DS. The distribution of each lifestyle factor was examined to assess the normality of the data and identify any outliers. Bivariate Pearson correlations, Kruskal‐Wallis tests, and point biserial correlations were used to examine the associations among lifestyle factors, socio‐demographics, and AD symptoms. Analyses were then conducted to determine whether engagement in leisure, employment, and physical activity moderated the association between amyloid age and AD‐related cognitive and functional declines. Lifestyle composite scores were calculated by taking the average of z scores from each study partner activity frequency score (leisure and employment activity) or step count (physical activity). Moderation analyses were run using the PROCESS macro for SPSS version 4.3.1. Cohen's *f* ^*2*^ and confidence intervals (CI) were calculated for each moderation effect for the lifestyle composite and each individual lifestyle component to determine if a specific activity was predominantly driving the effects observed using the lifestyle composite results. Cohen's *f* ^*2*^ were interpreted as small (0.02), medium (0.15), or large (0.35) (Cohen 1988).

## 2 Results

Amyloid age scores ranged from −12.93 to 8.62, indicating participants were estimated to be as far as 12 years from becoming Aβ + (i.e., Centiloids > 18) and others had been Aβ + for up to 8 years. Twenty‐one (33%) participants had positive amyloid age values, indicating those participants were Aβ+. NTG‐ESDS scores ranged from 0 to 46 and DSMSE scores ranged from 26 to 98 (means presented in Table 1). There was significant skewness for the NTG‐EDSD (2.67). Results remained consistent with log transformations of the NTG‐EDSD; therefore, raw scores are presented for interpretability.

Of the 25 cognitive leisure activities, participants engaged in 1 to 19 activities at least once a month (M = 12.62, SD = 3.92). The most common activities endorsed were listening to music (*n* = 60; 95.2%), helping with laundry (*n* = 58; 92.1%), eating at a restaurant (*n* = 53; 84.1%), and helping cook a meal (*n* = 52; 82.5%). Of the seven social leisure activities, participants engaged in 0 to 7 activities at least once a month (Figure 1). The most common activities endorsed were visiting with a friend or relative (*n* = 58; 92.1%) and talking with a friend or relative (*n* = 52; 82.5%). The sum of the frequency that adults participated in each activity (cognitive leisure frequency and social leisure frequency scores; means presented in Table 1) was highly correlated with the number of leisure activities (cognitive leisure *r* = 0.92, *p* < 0.001; social leisure *r* = 0.88, *p* < 0.001).

Of the employment activities, participants engaged in 0–9 of the nine activities. The most common employment activities were having discussions with others (discussion, training or learning something new, and assembling or sorting things) (Figure 2). The frequency score of employment activities was also considered (means presented in Table 1).

**FIGURE 3:**
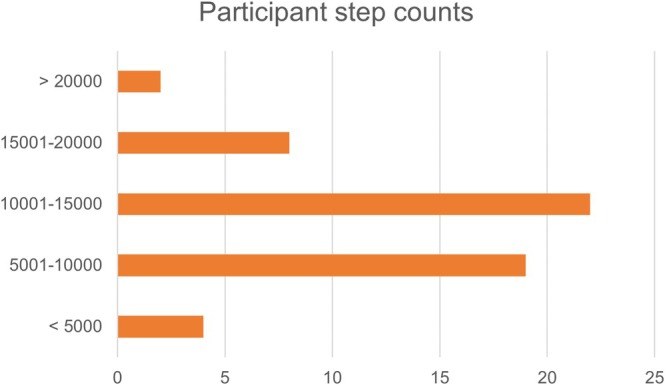
Number of adults with DS in each average step count category.

For the physical activity measure, participants wore the actigraphy watch for an average of 6.34 days. Eight (12.7%) participants were missing actigraphy data; three participants elected not to wear the wrist device due to sensory sensitivities, and five participants either removed the device prior to three days of data collection or the device did not properly record. There were no significant differences in age, dementia symptoms, or amyloid age between those with and without valid actigraphy data (*p* > 0.05). Daily step count ranged from 2312 to 21,203 (Table 1). Most individuals were in the range of 10,000–15,000 steps per day (Figure 3).

The association among frequency of cognitive leisure, social leisure, and employment activities, and average steps per day, socio‐demographics, and AD symptoms was examined (Table 2). Frequency of cognitive leisure was significantly positively correlated to frequency of social leisure, cognitively‐stimulating employment activities, and step count (*r =* 0.33 ─ 0.55). There was also a significant positive correlation between social leisure and employment activity frequency (*r =* 0.29). Individuals with mild ID participated in more cognitive leisure and had higher DSMSE scores compared to participants with moderate and severe/profound ID. Lifestyle composite scores were calculated by taking the average of z scores from each frequency activity rating (sum of study partner Likert scale ratings) or step count. Lifestyle composite z‐scores ranged from −1.89 to 1.54 (SD = 0.68).

**TABLE 2:** Bivariate Pearson correlations, Kruskal‐Wallis tests, and point biserial correlations among lifestyle factors, socio‐demographics, and AD symptoms.

	1	2	3	4	5	6^a^	7^b^	8	9
1.Cognitive leisure									
2.Social leisure	0.55***								
3. Employment activity	0.34**	0.29*							
4. Physical activity	0.33*	0.23	0.17						
5. Chronological age	−0.21	−0.14	−0.11	−0.20					
6. ID level^a^	7.75*	5.74	1.08	4.18	1.87				
7. Sex^b^	0.27*	0.07	0.24	−0.07	−0.08	3.62			
8. Amyloid age	−0.23	−0.12	−0.32*	−0.11	0.73***	2.47	−0.18		
9. NTG‐EDSD	−0.39**	−0.32*	−0.26*	−0.41**	0.49***	0.59	−0.07	0.44***	
10. DSMSE	0.53***	0.47***	0.26*	0.39**	−0.35**	19.30***	−0.32*	−0.37**	−0.51***

*** *p* < 0.001.

** *p* < 0.01.

* *p* < 0.05.

^a^ Kruskal‐Wallis test was used due to a categorical variable.

^b^ Point biserial correlations used due to dichotomous variable; ID = Intellectual disability; NTG‐EDSD = National Task Group–Early Detection and Screening for Dementia; DSMSE = Down Syndrome Mental Status Examination.

### 2.1 Moderation effect of lifestyle factors

**FIGURE 4:**
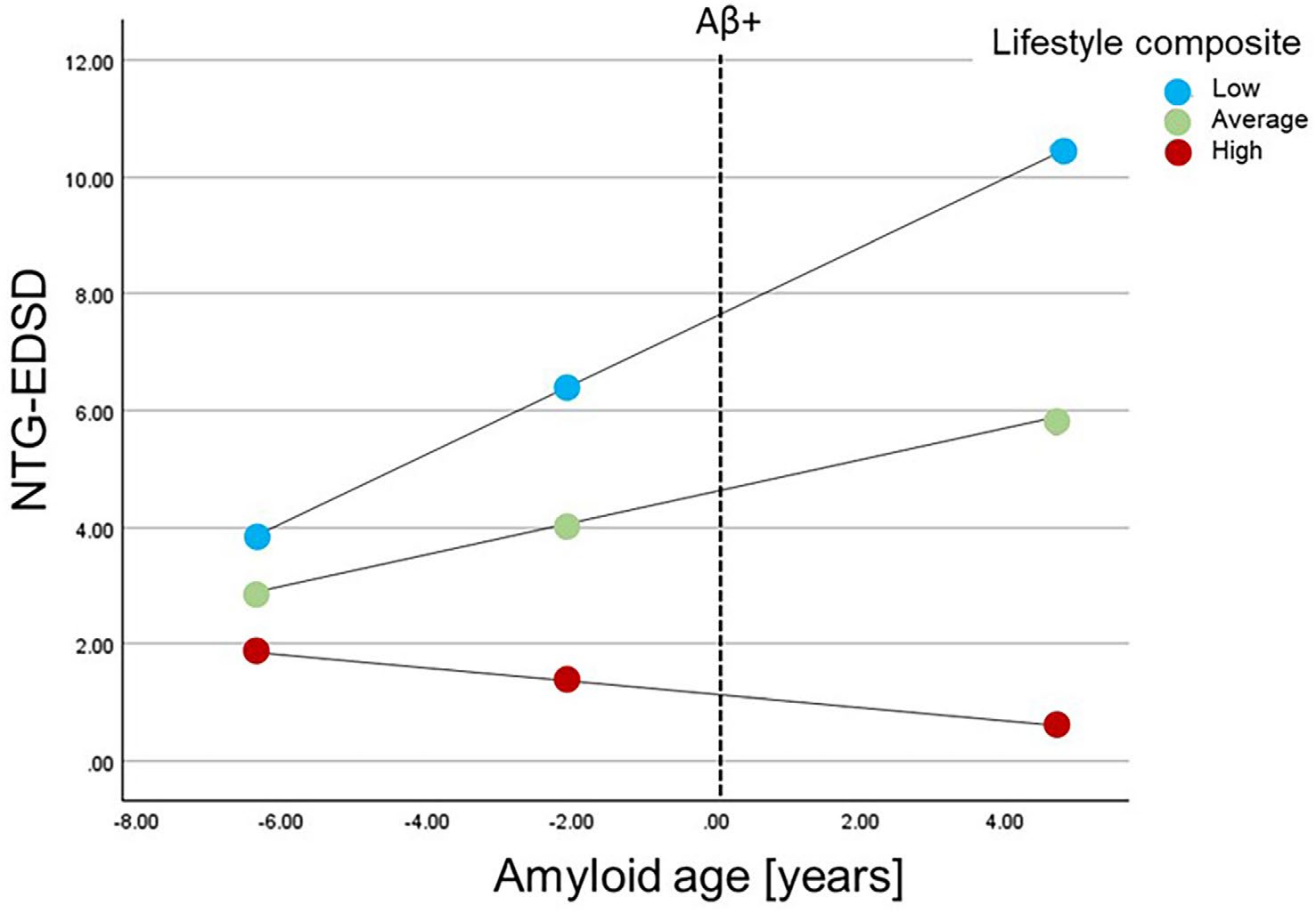
Moderation effect of lifestyle composite on the association between amyloid age and National Task Group‐Early Detection Screen for Dementia (NTG‐EDSD). Plot shows simple slopes at low (16th%), average (50th%), and high levels (84th%) of the moderator (lifestyle composite).

**FIGURE 5:**
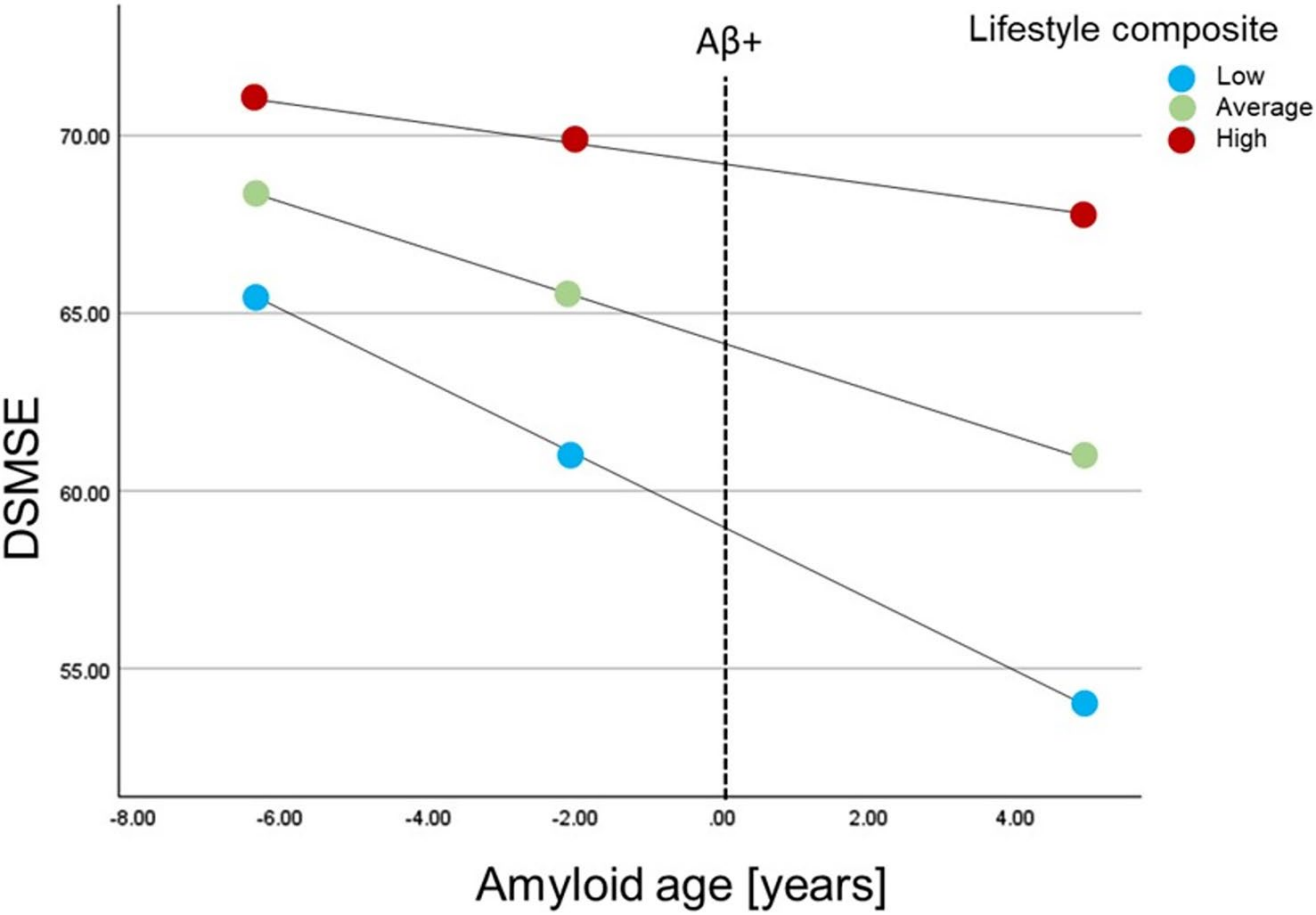
Moderation effect of lifestyle composite on the association between amyloid age and Down Syndrome Mental Status Examination (DSMSE). Plot shows simple slopes at low (16th%), average (50th%), and high levels (84th%) of the moderator (lifestyle composite).

Two regression models were run with amyloid age, the lifestyle composite and amyloid age x lifestyle composite as the predictors and AD symptoms (NTG‐EDSD or DSMSE) as the dependent variables (Table 3). ID level was also included as a control variable. There was a significant interaction of lifestyle composite X amyloid age on NTG‐EDSD, t (58) = −3.14, *p* = 0.003; and DSMSE, t (55) = 2.11, *p* = 0.04. Participants with a low lifestyle composite score experienced a greater effect of amyloid age on NTG‐EDSD, b = 0.59 [0.27, 0.90], t = 3.76, *p* < 0.001 (Figure 4) and participants with low and average lifestyle composite scores experienced a greater effect of amyloid age on DSMSE (b = −1.02 [−1.51, −0.54], t = −4.22, *p* < 0.001 and b = −0.65 [−1.15, −0.16], t = −2.65, *p* = 0.01; Figure 5).

**TABLE 3:** Lifestyle composite linear regression model results.

	NTG‐EDSD				DSMSE			
	B	SE (b)	*p*	95% CI	B	SE (b)	*p*	95% CI
ID level	−0.67	1.30	0.61	−3.28, 1.94	−11.78	2.01	< 0.001	−15.81, −7.74
Amyloid age	0.26	0.15	0.09	−0.05, 0.57	−0.68	0.24	0.007	−1.17, −0.19
Lifestyle composite	−4.98	1.42	< 0.001	−7.82, −2.14	7.60	2.25	0.001	3.09, 12.11
Amyloid age x lifestyle composite	−0.51	0.16	0.003	−0.83, −0.18	0.53	0.25	0.039	0.03, 1.03
	*R* ^2^ = 0.44				*R* ^2^ = 0.62			

Moderation analyses were run for each individual lifestyle factor (Table S1). Cohen's *f* ^*2*^ and confidence intervals (CI) were calculated for the lifestyle composite moderation effect and each lifestyle component individually (Table 4). The largest effect sizes were observed for physical activity (*f* ^*2*^ = 0.22 ─ 0.50).

**TABLE 4:** Cohen's *f* ^2^ effect size and confidence intervals (CI) for the interaction effect of each moderator.

	DSMSE			NTG‐EDSD		
	Interaction term β	95% CI of interaction term	*f* ^2^	Interaction term β	95% CI of interaction term	*f* ^2^
Lifestyle composite	0.53	[0.03, 1.03]	0.08	−0.52	[−0.84, −0.20]	0.18
Cognitive leisure only	0.33	[−0.05, 0.70]	0.06	−0.31	[−0.55, −0.06]	0.10
Social leisure only	0.47	[−0.01, 0.95]	0.07	−0.44	[−0.75, −0.12]	0.13
Employment activity only	0.44	[0.01, 0.87]	0.08	−0.37	[−0.65, −0.08]	0.11
Physical activity only	0.60	[0.17, 1.03]	0.17	−0.61	[−0.86, −0.37]	0.50

*Note:* DSMSE = Down Syndrome Mental Status Examination; NTG‐EDSD = National Task Group–Early Detection and Screening for Dementia.

## 3 Discussion

The goal of the current study was to investigate the moderation effect of a lifestyle composite made up of engagement in leisure, employment, and physical activity on the association between amyloid burden (assessed via amyloid age) and dementia symptoms in adults with DS. There was great variability in these lifestyle factors among the sampled adults with DS, as indicated by the range in the number of activities and frequency of engagement with the activities (Table 1; Figures 1, 2, 3). In support of our hypothesis, the lifestyle composite moderated the association between amyloid age and dementia symptoms. Specifically, adults with DS who had lifestyles involving greater engagement in cognitive and social leisure activities, employment activities that fostered learning and problem‐solving, and more physical activity evidenced fewer dementia symptoms given similar amyloid age values than adults with DS who engaged in fewer of these lifestyle activities. Specifically, adults with DS who had low lifestyle composite scores had a 0.59‐point increase on the NTG‐EDSD and a 1.02‐point decrease on the DSMSE for each one‐year increase in amyloid age. Although a one‐point increase or decrease on an informant‐based or direct clinical examination may seem modest, over time this may result in a shorter than average length of time to MCI and dementia for the group with low lifestyle composite scores.

Investigation into the effect sizes of each individual lifestyle factor indicated that physical activity had the greatest impact on the relation between amyloid age and dementia symptoms. Indeed, in contrast to our hypothesis, the moderating effect of physical activity alone (*f* ^2^ = 0.17–0.50) was stronger than the moderating effect of the lifestyle composite (*f* ^2^ = 0.08–0.18) on NTG‐EDSD and DSMSE scores. The protective effect of physical activity has also been highlighted in the general older adult population, with cardiovascular health mechanisms thought to drive resilience pathways (Erickson et al. 2022; Iso‐Markku et al. 2022); exercise is one specific recommendation for reducing dementia risk across the life course (Livingston et al. 2024). These results also corroborate prior findings showing that physical activity may help maintain cognitive functioning in the face of aging and early AD pathology in the DS populations (Fleming et al. 2021). Our findings of a medium‐sized resilience effect of physical activity suggest a need to focus on physical activity interventions either alone or as part of multicomponent lifestyle interventions to delay DSAD.

The moderation effect of the combined lifestyle composite was stronger than the individual effects of cognitive leisure, social leisure, and employment activities, which were each small‐sized (*f* ^2^ = 0.06–0.13). Although these individual activities had relatively small effect sizes, previous literature on the general older adult population has shown that engagement in multiple healthy lifestyle activities has synergistic effects on healthy cognitive ageing and reduced risk for AD (Dhana et al. 2020; Dhana et al. 2022; Sabia et al. 2009). Our findings are also consistent with previous research that identified social and cognitive leisure as buffering the negative effect of increasing amyloid burden on cognitive decline in DS (Mihaila et al. 2019). Additionally, these findings align with prior work showing that engagement in more complex paid or non‐paid employment activities is associated with less cognitive decline with age in adults with DS (Piro‐Gambetti et al. 2024). Future studies should investigate the specific resilience pathways (i.e., brain reserve, brain maintenance, or cognitive reserve) that drive these benefits. Current findings are based on cross‐sectional analyses; while only a handful of adults with DS had a clinical status of dementia (*n* = 4), it is still possible that early dementia symptoms caused changes in lifestyle factors. However, it is unlikely that these findings are primarily due to dementia‐related decline, as those with both high amyloid age and high lifestyle composite scores did not exhibit dementia symptoms. Additional follow‐ups on this cohort will provide more direct information on the effect of earlier lifestyle factors on later dementia symptoms and clinical status in adults with DS.

The idea that healthy lifestyle behaviors often co‐occur was supported in the current sample of adults with DS. With some exceptions, adults with DS who engaged in one healthy lifestyle activity were also more likely to participate in other beneficial lifestyle behaviors. There was a particularly strong association between engagement in cognitive and social leisure (*r =* 0.55), which may be attributed to the similar measurement methods, but could also signal that engaging in leisure activities in one domain promotes engagement in the other domain or that these activities often go hand‐in‐hand (e.g., doing a puzzle with a friend). Adults with DS who engaged in higher levels of cognitive leisure were also more likely to engage in higher levels of physical activity (*r* = 0.33) and employment engagement (*r =* 0.34), suggesting that cognitively stimulating activities may be supported by physical activity or employment engagement and vice versa. The variability observed also highlights the need to better understand the mechanisms behind these lifestyle variables in relation to amyloid age and cognition to identify the best ways to support a healthy lifestyle in adults with DS (Arenaza‐Urquijo and Vemuri 2020). Those with high activity may have more support from family or higher levels of independence. It is also possible that participation in a healthy lifestyle provides more opportunities to develop cognitive reserve, compensating for declines in cognitive abilities (Stern 2012).

### 3.1 Limitations and Future Directions

In terms of limitations, the study focused on cross‐sectional associations and did not assess the time‐ordered effect of lifestyle on the association between amyloid age and AD‐related cognitive decline. Future longitudinal research should include multiple follow‐ups to better understand the impact of a healthy lifestyle over time. Longitudinal work may also consider examining the lifestyle components separately to determine if engagement in particular activities has optimal attenuation of cognitive decline in a specific developmental period or age range. Effects of social determinants of health and also the degree of complexity of lifestyle activities should also be considered in future investigations of factors that may reduce dementia risk. This study also relied on study partner reports of engagement in leisure and employment activity, and future work would benefit from direct measures of daily activity and engagement in the community. Finally, the signal detection limits of PET scanners yield greater accuracy in estimating positive amyloid age values (years since becoming amyloid positive) than estimated years to amyloid positivity; as such, negative amyloid ages (i.e., prior to reaching CL 18) estimates should be interpreted with caution. Despite these limitations, this study provides initial evidence that engaging in more leisure activities that promote cognitive stimulation or social engagement, employment activities (paid or non‐paid) that promote problem‐solving and learning, and more physical activity may allow adults with DS to maintain their cognitive functioning for longer in the face of early AD pathology. Engaging in physical activity in particular may be a potential intervention target for promoting resilience to DSAD.

## Author Contributions

E.K.S. and S.L.H. wrote the original draft of the manuscript. E.K.S. and S.L.H. conceptualized the paper. Formal analysis and data visualizations were carried out by E.K.S. All authors reviewed the final manuscript. Funded was acquired by B.L.H., B.T.C., S.L.H., and the ABC‐DS (https://www.nia.nih.gov/research/abc‐ds#data).

## Ethics Statement

Procedures were approved by a central IRB (Advarra Pro00044843) and the University of Wisconsin‐Madison IRB (2020–1421).

## Consent

Consent was provided by the adult with Down syndrome or their legal guardian (with assent obtained from the adult with Down syndrome).

## Conflicts of Interest

B.L.H. receives royalties from two co‐authored books, is paid consulting fees from Patient‐Centered Outcomes Research Institute (PCORI) grant, received honoraria from the University of North Carolina and University of California Davis, and served on a data monitoring board for a Department of Defense funded grant. B.T.C. is on scientific advisory board for Alnylam Inc and has received equipment and materials from Avid Radiopharmaceuticals and Lantheus Inc. S.L.H. is a consultant for Ionis Pharmaceuticals and Alnylam Pharmaceuticals. The remaining authors declare no conflicts of interest.

## Supporting information


**Table S1.** Individual lifestyle factor linear regression model results.

## Data Availability

The data that support the findings of this study are openly available in ABC‐DS at https://www.nia.nih.gov/research/abc‐ds.
