# Supplementary material for: Lifestyle Composite and Resilience to Alzheimer's Disease Pathology in Down Syndrome
Source: J Appl Res Intellect Disabil. 2025 Aug 13;38(4):e70109. doi: 10.1111/jar.70109 (PMC12344623; doi:10.1111/jar.70109)
Supplement: Supplementary file 1 — Table S1. Individual lifestyle factor linear regression model results. [file JAR-38-e70109-s001.docx]

| Supplemental Table 1.  Individual lifestyle factor linear regression model results. | | | | | | | | |
| --- | --- | --- | --- | --- | --- | --- | --- | --- |
|  | **Cognitive leisure** | | | | | | | |
|  | NTG-EDSD | | | | DSMSE | | | |
|  | \| *B* \|  \|  \| \| --- \| --- \| --- \| | *SE(b)* | *p* | *95% CI* | \| *B* \|  \|  \| \| --- \| --- \| --- \| | *SE(b)* | *p* | *95% CI* |
| ID level | -.50 | 1.37 | .72 | -3.25, 2.25 | -11.92 | 2.03 | <.001 | -15.98, -7.85 |
| Amyloid age | .30 | .18 | .09 | -.06, .65 | -.70 | .26 | .01 | -1.23, -.17 |
| Cognitive leisure | -2.71 | 1.01 | .01 | -4.74, -.69 | 4.85 | 1.54 | .003 | 1.77, 7.93 |
| Amyloid age x cognitive leisure | -.30 | .13 | .02 | -.55, -.05 | .33 | .19 | .08 | -.05, .70 |
|  | R^2^ = 0.35 | | | | R^2^ = 0.60 | | | |
|  | **Social leisure** | | | | | | | |
|  | NTG-EDSD | | | | DSMSE | | |  |
|  | \| *B* \|  \|  \| \| --- \| --- \| --- \| | *SE(b)* | *p* | *95% CI* | \| *B* \|  \|  \| \| --- \| --- \| --- \| | *SE(b)* | *p* | *95% CI* |
| ID level | -.85 | 1.36 | .54 | -3.58, 1.88 | -11.61 | 2.05 | <.001 | -15.71, -7.51 |
| Amyloid age | .43 | .16 | .008 | .11, .74 | -.89 | .24 | <.001 | -1.36, -.42 |
| Social leisure | -3.38 | 1.03 | .002 | -5.44, -1.32 | 5.29 | 1.59 | .002 | 2.11, 8.48 |
| Amyloid age x social leisure | -.44 | .16 | .007 | -.75, -.12 | .47 | .24 | .05 | -.003, .95 |
|  | R^2^ = 0.35 | | | | R^2^ = 0.59 | | | |
|  | **Employment activity** | | | | | | | |
|  | NTG-EDSD | | | | DSMSE | | |  |
|  | \| *B* \|  \|  \| \| --- \| --- \| --- \| | *SE(b)* | *p* | *95% CI* | \| *B* \|  \|  \| \| --- \| --- \| --- \| | *SE(b)* | *p* | *95% CI* |
| ID level | .25 | 1.39 | .86 | -2.54, 3.04 | -13.80 | 2.11 | <.001 | -18.03, -9.58 |
| Amyloid age | .45 | .17 | .01 | .11, .79 | -1.01 | .26 | <.001 | -1.53, -.49 |
| Employment | -1.80 | 1.08 | .10 | -3.96, .37 | 1.21 | 1.65 | .47 | -2.11, 4.52 |
| Amyloid age x employment | -.37 | .14 | .01 | -.66, -.08 | .44 | .22 | .04 | .003, .87 |
|  | R^2^ = 0.29 | | | | R^2^ = 0.54 | | | |
|  | **Physical activity** | | | | | | | |
|  | NTG-EDSD | | | | DSMSE | | |  |
|  | \| *B* \|  \|  \| \| --- \| --- \| --- \| | *SE(b)* | *p* | *95% CI* | \| *B* \|  \|  \| \| --- \| --- \| --- \| | *SE(b)* | *p* | *95% CI* |
| ID level | .55 | 1.29 | .67 | -2.05, 3.14 | -12.99 | 2.20 | <.001 | -17.43, -8.56 |
| Amyloid age | .22 | .15 | .15 | -.08, .52 | -.83 | .26 | .002 | -1.35, -.31 |
| Physical activity | -3.56 | .87 | <.001 | -5.32, -1.81 | 4.67 | 1.51 | .003 | 1.63, 7.71 |
| Amyloid age x physical activity | -.63 | .12 | <.001 | -.88, -.38 | .60 | .21 | .007 | .17, 1.03 |
|  | R^2^ = 0.53 | | | | R^2^ = 0.60 | | | |

Note. DSMSE =Down Syndrome Mental Status Examination; NTG-EDSD = National Task Group – Early Detection and Screening for Dementia.
